# Refocusing public health training on effective leadership and communication skills to increase efficacy

**DOI:** 10.3389/fpubh.2025.1601444

**Published:** 2025-06-16

**Authors:** Sharia Phillips, Jasmine Hamilton, Wayne McCullough, Darline El Reda, Constance Currier, John Clements

**Affiliations:** Department of Public Health, Michigan State University, East Lansing, MI, United States

**Keywords:** student training, public health, leadership and communication, ecological leadership, community partnerships and collaborations

## Abstract

Public health (PH) practitioners recognize the importance of cultivating future leaders, especially as the value of public health is increasingly scrutinized nationally. To address this scrutiny, the PH workforce must demonstrate through its leadership adept communication skills and informed conversations leading to decisions based on science. The Master of Public Health and other programs can play a crucial role in the development of these skills through training and experiential opportunities allowing students to practice and enhance their leadership and communication efficacy. This paper addresses how we start rethinking the way we train the next generation of PH professionals. It also focuses on leadership and effective communication and how we can upskill those currently in the profession to address communication challenges and therefore move the field forward.

## Introduction

Public health (PH) has come under increased scrutiny in the national discourse. Much of the recent PH narrative is rooted in communication disconnects and disinformation. According to a 2024 qualitative study conducted by the de Beaumont Foundation and Communicate Health, US adults have minimal knowledge of the PH workforce’s role in society and of the work they do (1). This finding emphasizes a PH communication gap in how often we tell our story and underscores a PH dilemma. If the public does not know what we do or who we are, they will be less likely to consider our guidance and preventative efforts to improve their health and wellbeing. Our messages and outreach will not resonate, and guidance will not be followed. Klerings et al. (2) assert the increase in online health sites and resources has led many to become over-saturated and overwhelmed with information which inhibits their ability to sift through the facts and discern what is being said and how it is being supported. We argue that today the available number of online health resources has grown exponentially, so much so, that people are still experiencing information fatigue but are more willing to piece together snippets of facts to problem-solve and to find answers. Part of the disconnect is those overwhelmed look to a trusted agent to distill the many perspectives—regardless of the expertise of the agent. At first glance, this self-informed era seems laudable, however our challenge becomes the extent to which that information is being accurately consumed and critically evaluated in the absence of PH guidance. As the public becomes more reliant on information clicks to form opinions and make decisions, it becomes increasingly important for PH leaders to sharpen their communication acumen and re-engage the public.

Undoubtedly, future public health leaders will also continue to grapple with this PH information disconnect as well as with this self-taught era. Master of Public Health Programs can play a vital role in the training of future PH leaders; one that rethinks the scope of how leadership traits can provide different avenues for students to develop their communication skills. This paper addresses how we must start training the next generation of PH professionals with a renewed emphasis on developing leadership traits that foster enhanced communication skills and how we can upskill those currently in the profession, so they can tackle these communication challenges and advance the field.

## Current public health landscape and need to adjust leadership approaches

To translate and effectively communicate scientific information in ways that make sense to and have impact upon the public(s), PH leaders need to embrace their role in the health literacy of individuals and communities. The US Department of Health and Human Services’ Healthy People 2030 initiative expanded the scope of health literacy to include the health field’s responsibility to improve how they communicate with the public (3). This added definition underscores the ownership health organizations must have to improve individuals’ health literacy. It also calls for PH leaders to rethink how they engage the public including how relationships are fostered and how ongoing outreach and tailored guidance can happen as a result.

Preparing future public health leaders to hone their skills to address the communication challenges calls for a broader approach to understanding how leadership traits and practices can lead to enhanced communication skills. Magaña and Biberman (4) argue the next generation of public health workers should be equipped with people-oriented traits that foster a collective human spirit focused on how to problem solve, collaborate, listen to, adapt with, and embrace interacting with diverse populations. Because public health leaders operate in complex environments compromised by diverse groups these skills are essential. We must therefore adopt approaches that prepare leaders to pivot and recognize how their engagement with the public and different sectors regarding political and social climate will change.

To this end, we present both Situational and Ecological leadership together and suggest that leaders cannot operate in vacuums. Both define traits to engage with individuals and groups to which they accordingly adapt leadership and communication styles based on the needs of the population to achieve maximal impact.

### Situational leadership

Hersey’s and Blanchard’s situational leadership model places emphasis on leaders’ ability to recognize that each situation is unique and asks leaders to modify their behaviors to better meet the needs of those who work for them. These behaviors include Directing, Coaching, Supporting, and Delegating (5) and represent tailored guidance that rests on the belief that situational leaders know how to assess workers’ ability or “readiness” to complete job tasks successfully. They do not practice a singular philosophy or communication style and expect others to conform. Similarly, The Center for Leadership Studies (6), asserts situational leaders know how to engage with others to understand their assigned task efficacy; understand when to change course to ensure a task aligns with a person’s strengths; know how to communicate with purpose and direction; and recognize how to create a path to task success for everyone.

### Ecological leadership

According to Wielkiewicz and Stelzner (7), ecological leadership acknowledges how understanding the inner workings of an organization and of the environment which they refer to as “ecological systems” (p. 330) play a key role in leaders’ ability to be effective. This is where the application of style and understanding of the “environment” become so important. Knowing how to lead within these ecological systems influences the extent to which leaders can move organizations forward. Wielkiewicz and Stelzner’s (7) ecological approach also discusses traits leaders should practice such as: building ongoing relationships with the communities they serve and within the organizations they work; balancing divergent perspectives to promote change; adjusting based on the current workplace, social, and political environments; and embracing input including suggestions and constructive insight.

## Connections between situational and ecological leadership

Both situational and ecological leaders possess traits and characteristics that support their connections to being adept communicators. The different behaviors situational leaders use to help increase workers’ task efficacy and determine what level of guidance to employ overlap with ecological leadership. Both focus on people skills and relationships. To employ the appropriate behavior related to employees’ readiness to take on an assignment, situational leaders rely on their communication skills to know who the employee is and how best to offer guidance. Similarly, for ecological leaders to be effective, they rely on knowing how to engage others so they can understand the relationship between the sum of an organization’s parts and of the environment. This conceptual awareness enables ecological leaders to communicate with a broad range of groups in ways that will move the organization forward. The overlaps in these models evolve around knowing how to maximize interactions with others to facilitate individual, group, and organizational impact.

We know future public health leaders should be not only technically skilled but also skilled communicators and advocates who recognize how an environment can influence what needs to be communicated and how it should be done. Both situational and ecological leadership models emphasize the value of seeing the whole picture, seeking to understand the context and then using that knowledge to approach conversations based on the situation.

## Leadership traits and their connections to communication skills

In an era of misinformation, effective communication is not only a skill but a critical tool for encouraging and restoring public trust. Effective communication is central to public health leadership. It involves more than just disseminating information; it requires translating technical knowledge into accessible, actionable messaging. As a reflexive process, engaging others also requires understanding how to meet people where they are by becoming active listeners. This builds trust and ensures that diverse perspectives are acknowledged. Situational and ecological leaders practice these traits which creates an atmosphere for this reflexive process to occur.

To further develop the field, future public health leaders’ ability to facilitate in-depth discussions and to engage others in meaningful ways requires active listening. Successful engagement depends on a blend of strategic communication, evidence-based arguments, and storytelling to build emotional connections. Those connections form from feeling heard. Employing adaptability supports the communication process and helps leaders navigate the political landscapes to ensure conversations are reflexive. According to Olsen and Bastholm (8) “adaptive decision making” (p. 5) is a crucial skill for public health leaders. Whether addressing emerging infectious diseases, climate change impacts, or shifts in funding priorities, adaptability enables leaders to effectively interact with others in increasingly uncertain contexts. This skill involves staying informed, embracing innovation, and adjusting messages to improve information consumption.

## Our case study example: student advisory board—a framework for honing leadership and communication skills

As an online program, we know the virtual space offers unparalleled flexibility and accessibility. Identifying how to increase students’ understanding of how leadership traits can improve communication skills presents a unique opportunity. The absence of a shared physical or synchronous virtual environment can alter the ways communication skills occur. To address these challenges, we developed a virtual student advisory board. This board provides a framework for students to increase their working leadership knowledge and deepen their communication skills. It creates experiential opportunities in real time where students take leadership roles and work with diverse groups ranging from MPH faculty and staff, other university offices, community members, and public health organizations to plan public health activities.

### SAB framework

The Student Advisory Board (SAB) is an elective board with an average time commitment equal to a few hours a month unless students chooses to serve on a committee at which the level of commitment will vary. Students in leadership roles, for example (executive board members, committee chairs, special-event-point person), devote more time to plan, implement, and evaluate activities. Though membership fluctuates based on incoming students and those who have graduated, around 20–30% of the student body chooses to be involved in the SAB. While faculty and program staff are involved, the SAB is primarily student lead and run—one faculty or program staff member attend the bi-weekly meeting for guidance as needed, though more interaction is readily available based on the board’s needs.

**Figure 1:**
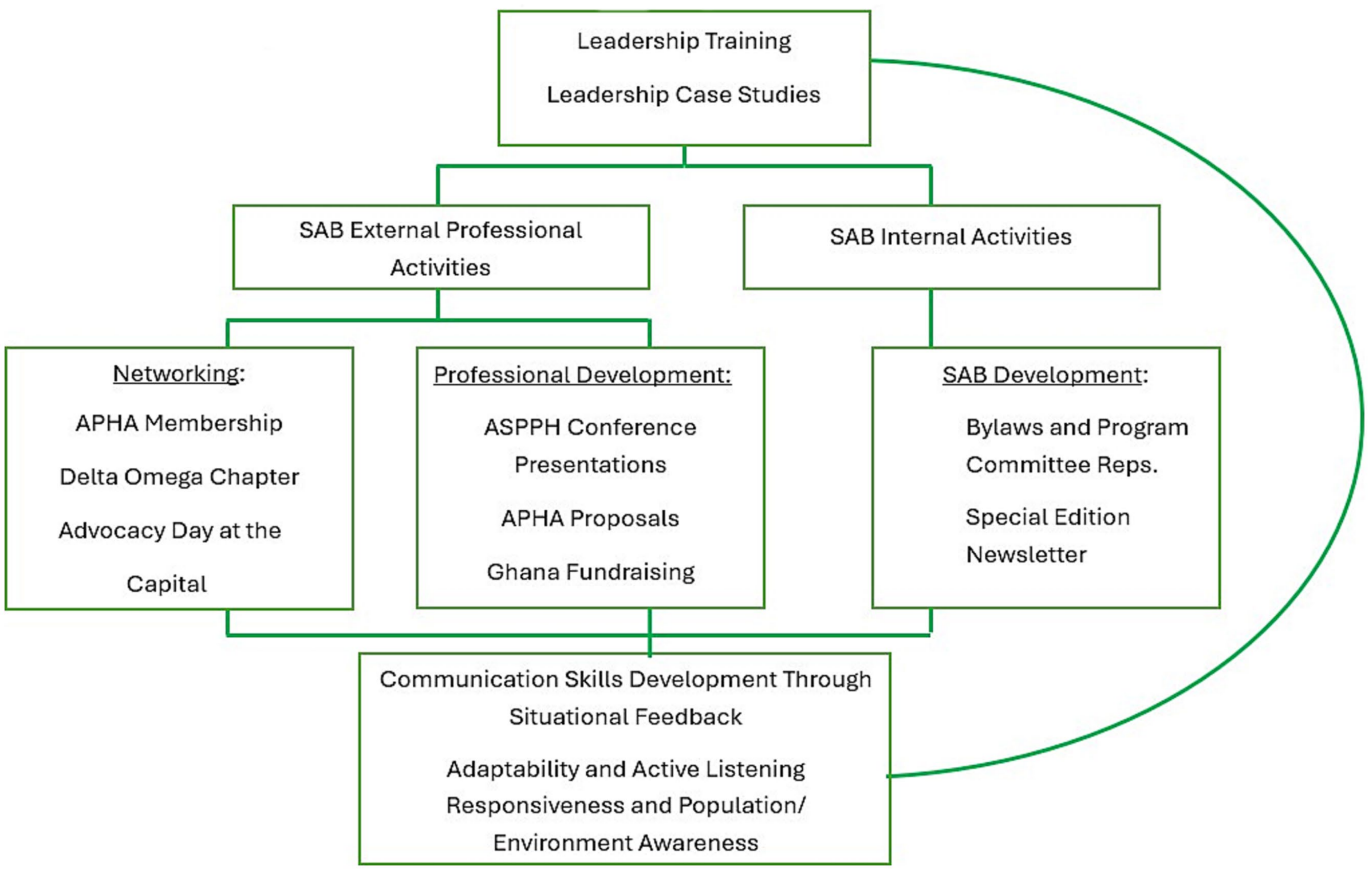
The connection between leadership and communication development: situational opportunities for demonstrating ecological and situation leadership: The Michigan State University SAB (adapted from The Hill Model for Team Leadership [(9), p. 367]).

The SAB Framework provides external professional activities that foster experiential leadership opportunities through partnerships, professional development and networking. SAB students also regularly participate in internal activities which require continual discussions during our Zoom board meetings. All of these are dependent upon the student leaders’ ability to engage with others and communicate in ways that reach different audiences and consider different perspectives. However, while these SAB activities are voluntary, all students in the Master of Public Health program are required to take a course titled ‘Principles of Public Health Leadership’ which focuses on topics such as: theories, styles, policy development, community, collaboration, advocacy, and cross-cultural issues (see Figure 1).

#### SAB external professional activities

Our program covers a two-year APHA (American Public Health Association) membership for students. This membership provides an avenue for SAB members to apply for PH grants designed for student initiatives, present at conferences, and attend webinars. Champions for Climate Change, an APHA student grant opportunity came from that membership. SAB students wrote a grant focused on air quality awareness and became one of the five funded proposals. Engaging with state political leaders during a sponsored Advocacy Day at the capital and collaborating with non-profit organizations to host fundraising events are other SAB partnered events.

SAB leaders facilitate cross-sectional projects too like career events including resume and cover letter panels, and mock interview sessions in partnership with the University Career Network Services and with the hiring managers from the Michigan Public Health Institute. These partnerships create opportunities for students to moderate discussions around careers and jobs within the PH field and to prepare for job interviews.

#### SAB internal activities

SAB students hold Zoom meeting every 2 weeks. Multiple conversations each semester have led to board bylaws, a voting structure for leadership positions and program committee representatives, town halls, and a program newsletter. Two of the newsletters that garnered specific discussion and debate were special issues focused on Roe vs. Wade and gun violence. These internal activities require leadership and communication. Additionally, the SAB developed a ‘Faculty 30’ webinar series where MPH program faculty give relaxed presentations on their research interests which creates opportunities for student to connect their interests with faculty who have similar research agendas.

#### Situational feedback and communication skills development

Most SAB activities present recurring opportunities for students to engage in constructive feedback loops that enhance their leadership and communication skills. Feedback is integrated across multiple dimensions—internally within the board, from faculty and staff, and externally from public health organizations and professional networks. For example, when SAB members facilitate events or serve on committees, they receive real-time feedback from participants, faculty, and staff, allowing them to refine their public speaking, adaptability, and problem-solving skills.

Additionally, SAB provides a structured yet flexible environment for faculty-student feedback exchanges beyond traditional course evaluation. This direct dialogue fosters more meaningful mentorship and professional development, equipping students with the confidence and skills to articulate their ideas, advocate for initiatives, and engage in reflective leadership practices.

Externally, SAB members interact with organizations such APHA when submitting conference proposals, participating in networking events, or applying for public health grants. These experiences expose them to professional critiques, which serve as valuable learning moments, reinforcing the importance of effective communication, resilience, and adaptability in leadership. By navigating feedback in these various contexts, students develop a nuanced understanding of how to assess, interpret, and apply constructive criticism-an essential skill for future public health leaders who must communicate effectively in diverse and often challenging environments.

## Discussion

### Leadership and communication are keys to regaining the public(s) trust

Earlier we discussed the problematic climate of information dissonance facing public health leaders. Delivering the message is only a part of protecting the health of individuals and communities. Public trust in public health has been undermined by widespread misinformation and growing skepticism regarding scientific guidance. Effective leadership is not just about managing teams or implementing policies—it is also about fostering trust through clear, consistent, and accessible communication. As previously discussed, the current landscape of information dissonance presents a critical challenge for public health leaders. Situational and ecological leadership models provide pathways for improving engagement with diverse audiences by emphasizing adaptability and relational understanding.

To rebuild trust, public health leaders must embrace their role as communicators and trusted messengers. This means shifting from simply delivering messages to actively engaging with the communities they serve. Trust is not built solely on the accuracy of the information provided but also on the way it is conveyed. Leaders who prioritize transparency, empathy, and responsiveness are more likely to foster trust and encourage the adoption of public health guidance. The next generation of public health professionals must be trained to understand these nuances, ensuring that they can navigate complex information landscapes effectively.

### Situational and ecological leadership emphasize how to communicate with different audiences in ways that have maximal impact

Employing leadership traits hones leaders’ communication acumen and creates opportunity for more productive conversations with diverse groups. Both situational and ecological leadership emphasize the importance of tailoring communication strategies to different audiences. Effective leaders recognize that a one-size-fits-all approach to communication is insufficient in a diverse and evolving public health landscape. Instead, they assess the environment, understand the needs of their audience, and adjust their messaging accordingly.

Situational leadership requires leaders to modify their communication styles based on their audience’s level of knowledge, engagement, and readiness to act. This approach helps leaders guide individuals and groups toward informed decision-making while addressing any barriers to understanding. Similarly, ecological leadership acknowledges the interconnectedness of various stakeholders and the environments in which they operate. Leaders who adopt this approach are better equipped to navigate political, social, and organizational complexities, ensuring that public health messages are contextually relevant and impactful.

By integrating these leadership frameworks into public health training, future leaders can develop communication strategies that are not only evidence-based but also culturally and socially attuned. This integration enhances their ability to engage diverse populations, build consensus, and drive meaningful public health interventions.

### Listening as a communication skill drives effective leadership—we need to dramatically hone these skills

Engaging with others relies on more than a verbal exchange. As we mentioned before, the communication process is a transactional process. A core component of this process centers on listening.

Listening is an essential, yet often underappreciated, component of effective communication. Public health leaders must move beyond simply disseminating information to actively engaging in two-way dialogues with their communities. Active listening fosters trust, enhances understanding, and ensures that public health messages resonate with the intended audience.

Engagement is a transactional process—leaders must be as receptive as they are expressive. Listening involves not only hearing words but also understanding concerns, recognizing cultural contexts, and responding appropriately. By honing active listening skills, public health leaders can address misinformation, clarify misunderstandings, and create communication pathways that are more inclusive and effective.

The Student Advisory Board (SAB) serves as a valuable case study in applying leadership and communication principles in real-world settings. Through partnerships, professional development, and committee representation, SAB members gain firsthand experience in leadership roles that require them to listen, adapt, and communicate effectively. Their experiences demonstrate that leadership training should prioritize the development of strong listening skills alongside traditional leadership traits.

Regaining public trust, maximizing communication impact, and fostering active listening are critical components of effective public health leadership. By integrating situational and ecological leadership principles into public health training, we can equip the next generation of leaders with the skills necessary to navigate complex communication landscapes.

## Data Availability

The original contributions presented in the study are included in the article/supplementary material, further inquiries can be directed to the corresponding author.
